# Reaching Nepal’s mothers in time

**DOI:** 10.2471/BLT.16.030516

**Published:** 2016-05-02

## Abstract

Women’s chances of survival during pregnancy and childbirth have greatly improved in Nepal. Sophie Cousins reports.

Standing at a community health post and looking around the valley, Laxmi Tamang, a nurse and public health expert from the Midwifery Society of Nepal, points to the other side of the mountain.

“See that, all the way over there? That’s all the same district. If a woman is in labour, how on earth will she get here?”

The Midwifery Society of Nepal is running an outreach health camp in Nuwakot, 75 km from Nepal’s capital of Kathmandu, after much of the district’s infrastructure was damaged by the April 25 earthquake last year.

But for expectant mothers living in remote parts of the district – where there are few if any roads – accessing this midwifery care, especially in emergencies, is difficult – if not impossible.

**Figure:**
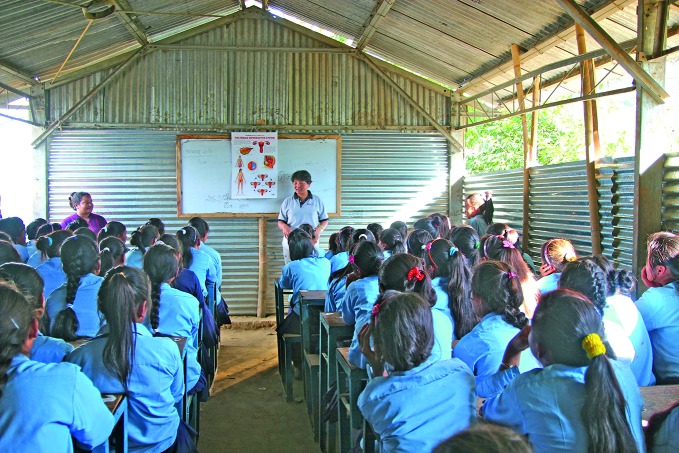
An antenatal check-up of an expectant mother at Tribhuvan University Teaching Hospital in Kathmandu, the Nepalese capital

WHO/Sophie Cousins

Nepal, a landlocked country in south Asia and home to some of the world’s tallest peaks, is considered to be a success story when it comes to improving the survival of its mothers in pregnancy and childbirth.

Between 1990 and 2015, the country reduced its maternal mortality ratio by 71% from 901 deaths per 100 000 live births to 258 – just four percentage points short of millennium development goal (MDG) 5: to reduce the maternal mortality ratio by three quarters during that time period.

For this achievement, Nepal, which has been recovering from a decade-long civil war between 1996 and 2006, was selected out of 49 least-developed countries by the United Nations in 2010 to receive an award for its leadership, commitment and progress towards the achievement of MDG 5.

Experts attribute Nepal’s success in improving maternal health to several factors, including free delivery services, transport incentives, access to safe delivery services and the use of the oral drug misoprostol for preventing postpartum haemorrhage during home deliveries or on the way to a health facility.

A study published in July 2014 in this journal on the success factors for women’s and children’s health shows that half the reduction in child mortality in low- and middle-income countries since 1990 resulted from health sector investments while the rest could be attributed to investments made in sectors outside health, such as education and road infrastructure.

Over the past decade, more women in Nepal are giving birth in a health facility than at home – another factor that has reduced the risk of postpartum haemorrhage (loss of blood following the birth), which is the leading cause of maternal deaths worldwide.

In 2006, homebirths accounted for about 81% of births in Nepal. In 2011 that figure dropped to 63%, according to the Nepal Demographic and Health Survey reports for 2006 and 2011 respectively.

 “Twenty-five years ago in the hilly regions of Nepal women had to deliver at home and maternal mortality was very high,” says Dr Kiran Regmi, chief specialist at the health ministry.

“Twenty-five years ago in the hilly regions of Nepal women had to deliver at home and maternal mortality was very high.”

Kiran Regmi

“Now, caesarean sections are available, even in remote areas. There are birthing centres and health posts across the country, where safe delivery is assisted by skilled birth attendants,” says Regmi, a former director of the health ministry’s family health division.

In 2005, Nepal’s government started providing stipends to help cover the transport costs for women to reach accredited birthing facilities and four years later added free delivery services to expectant mothers under its Safe Motherhood Programme.

The programme extended these benefits in 2011 by giving women US$ 5 if they came to a health facility for four antenatal check-ups during their pregnancy.

“In the past, most women faced a huge economic barrier,” says Dr Meera Upadhyay, an obstetrics and gynaecology chief consultant at Paropakar Maternity and Women’s Hospital in Kathmandu.

“We found out that women do come to the hospital to give birth when delivery services are free of charge,” she says, adding: “The policy has emphasized the availability of services for everyone: for the poor, the uneducated and for people living in remote areas.”

According to the head of Nepal’s Safe Motherhood Programme, Dr Shilu Aryal, in areas that are inaccessible by road, women are often carried on stretchers and, in emergencies, airlifting is an option in five regions of the landlocked country.

But for some mothers, getting to a health facility is still virtually impossible and others still prefer to give birth at home for cultural reasons, Aryal says.

Another factor that has also reduced maternal mortality is the legalization of abortion in 2002. There are now 500 registered sites providing safe terminations across the country, Aryal says.

“When I worked in maternity hospitals in the 1990s, women used to go to quacks or [unlicensed] medicine shops to get something to terminate unwanted pregnancies and sometimes suffered terrible consequences.

“They didn’t tell us what they’d done. They just came to the hospital and died,” Aryal says, adding that these services are available in all 75 districts up to 12 weeks of gestation, and in 42 of those districts trained auxiliary nurse midwives provide terminations at community-level health institutions up to nine weeks.

Regmi says that this legislation, together with the provision of free family planning, has been crucial in reducing the number of deaths from the consequences of unwanted pregnancies in Nepal.

In the absence of qualified midwives, women in Nepal rely on more than 50 000 female community health volunteers and more than 6000 skilled birth attendants: auxiliary nurse-midwives with 18 months training in maternal and child health care, as well as doctors and nurses.

The volunteers educate women on sexual and reproductive health matters, provide them with family planning advice and distribute oral contraceptives and condoms, and they refer expectant mothers to health facilities.

While efforts to combat postpartum haemorrhage have traditionally focused on the use of the injectable drug oxytocin, as the preferred uterotonic drug for facility-based births; back in 2005, when about 80% of births took place at home, Nepal’s government approved the use of the oral drug misoprostol.

Aryal explains that female community health volunteers provide misoprostol to women who deliver in rural areas, at home or on the way to a health facility to prevent postpartum haemorrhage.

However, while there have been huge improvements in reducing maternal deaths, there is still more work to be done.

In recognizing the country’s desperate need for midwives and their key role in reducing maternal mortality, three of Nepal’s universities plan to start offering a Bachelor of Midwifery course later this year.

“We have a huge gap both in the number and quality of staff providing maternity services in remote areas,” Upadhyay says.

To bridge this gap, incentives are needed to get midwives to work in the areas where they are needed.

“We need local people who go away to study and who then come back and work in their communities. We need to give more opportunities to people from remote areas to retain them and provide continuous service,” Upadhyay says.

Tamang, a qualified nurse, is the co-founder of the APS Birth Centre, the only independent birthing centre managed privately by a nurse-midwife in Nepal. Much of her work focuses on health education and advocating for the formal recognition of midwives as a profession in Nepal.

“In Nepal, girls do not have the right to put their critical thoughts to parents, relatives, teachers or anyone else,” Tamang says to a group of school-age girls in Nuwakot district.

**Figure:**
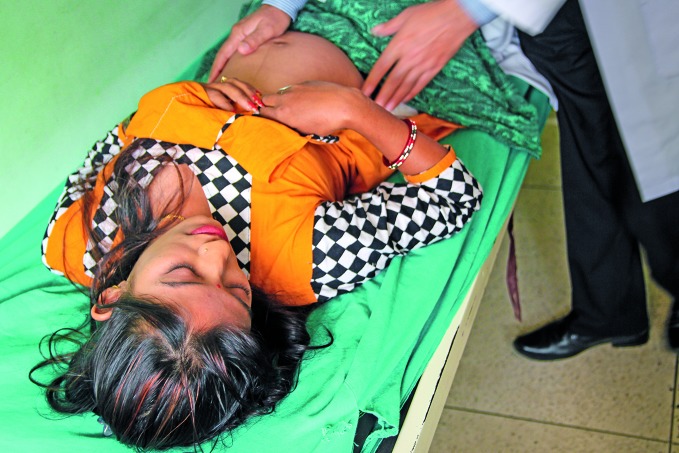
Laxmi Tamang, nurse and public health expert, talking to school students in Nuwakot district about early marriage and contraception

WHO/Sophie Cousins

She talks to them of the perils of adolescent pregnancy and early marriage, noting that while it is illegal to marry before the age of 18 in Nepal, one in four girls is married off before then.

“Every girl should be educated about early marriage and teenage pregnancy … because it will shape their future,” says Tamang, who is dedicated to empowering girls through health and sex education.

“Every girl should be educated about early marriage and teenage pregnancy … because it will shape their future.”

Laxmi Tamang

Tamang believes that her country can do a great deal more and welcomes the courses in professional midwifery that start this year in Nepal. “We should learn the lessons of Sri Lanka and other south Asian countries that have trained and deployed professional midwives to provide community-based primary health-care services”.

